# DynMultiDep: A Dynamic Multimodal Fusion and Multi-Scale Time Series Modeling Approach for Depression Detection

**DOI:** 10.3390/jimaging12010029

**Published:** 2026-01-06

**Authors:** Jincheng Li, Menglin Zheng, Jiongyi Yang, Yihui Zhan, Xing Xie

**Affiliations:** 1 School of Artificial Intelligence and Computer Science, Nantong University, Nantong 226019, China; 2330110412@stmail.ntu.edu.cn (J.L.); 2230110478@stmail.ntu.edu.cn (M.Z.); yjy2004@stmail.ntu.edu.cn (J.Y.); zyhntu@stmail.ntu.edu.cn (Y.Z.); 2 Engineering Training Center, Nantong University, Nantong 226019, China

**Keywords:** dynamic multimodal fusion, multi-scale time series modeling, intelligent depression detection, dynamic routing mechanism, resource-aware optimization

## Abstract

Depression is a prevalent mental disorder that imposes a significant public health burden worldwide. Although multimodal detection methods have shown potential, existing techniques still face two critical bottlenecks: (i) insufficient integration of global patterns and local fluctuations in long-sequence modeling and (ii) static fusion strategies that fail to dynamically adapt to the complementarity and redundancy among modalities. To address these challenges, this paper proposes a dynamic multimodal depression detection framework, DynMultiDep, which combines multi-scale temporal modeling with an adaptive fusion mechanism. The core innovations of DynMultiDep lie in its Multi-scale Temporal Experts Module (MTEM) and Dynamic Multimodal Fusion module (DynMM). On one hand, MTEM employs Mamba experts to extract long-term trend features and utilizes local-window Transformers to capture short-term dynamic fluctuations, achieving adaptive fusion through a long-short routing mechanism. On the other hand, DynMM introduces modality-level and fusion-level dynamic decision-making, selecting critical modality paths and optimizing cross-modal interaction strategies based on input characteristics. The experimental results demonstrate that DynMultiDep outperforms existing state-of-the-art methods in detection performance on two widely used large-scale depression datasets.

## 1. Introduction

Depression, one of the most common mental illnesses, is typically characterized by physiological symptoms such as weight loss, and insomnia and, in severe cases, may lead to suicide or substance abuse [1,2,3,4,5,6]. In recent years, the global prevalence of depression has been steadily increasing. According to the World Health Organization (WHO), the number of people suffering from depression worldwide exceeded 380 million in 2023 [7]. In China, according to the “2023 China Mental Health Blue Book,” the number of depression patients has reached 95 million. However, the diagnosis rate for depression in China is only 9.5%, meaning the vast majority of patients do not receive timely diagnosis and treatment, posing a significant challenge to mental health care [8,9,10,11]. Currently, the diagnosis of depression still relies on manual assessments, which not only can lead to misdiagnosis but also incur high diagnostic costs [12,13,14]. With the growing number of patients and the limitations of manual diagnosis, there is an urgent need to develop more efficient and intelligent depression detection systems to address this increasingly severe public health issue.

Depression detection based on multimodal data has become one of the key areas of current research, aiming to improve diagnostic efficiency and accuracy [15,16,17,18,19]. Traditional depression detection methods primarily rely on single-modal data, such as text, speech, or physiological signals. While these methods perform well in specific scenarios, they often struggle to comprehensively capture the emotional and behavioral changes of patients when faced with the complexity of depression symptoms [20,21,22]. Depression symptoms typically exhibit multidimensional and multi-scale characteristics, including both long-term trends and short-term fluctuations [23]. Therefore, effectively integrating multimodal data and extracting subtle changes related to depression has become a core challenge in current research.

Recent advances in multimodal fusion have significantly improved performance in various perception tasks, particularly by leveraging complementary information from different data sources. Early works primarily focused on image and video fusion techniques, aiming to combine spatial and temporal features effectively. More recent studies have explored the integration of vision and language modalities, as well as robust fusion strategies for handling noise and correlations across modalities. Notable contributions include a unified solution to video fusion through multi-frame learning and benchmarking, image fusion guided by vision-language models, and equivariant multi-modality image fusion techniques. In addition, denoising diffusion models have been applied to improve multi-modality fusion quality, and correlation-driven dual-branch feature decomposition has demonstrated effectiveness in capturing complementary information. These studies collectively highlight the importance of designing fusion methods that can handle heterogeneous data while preserving critical information across modalities.

Existing multimodal depression detection methods have benefited from static fusion schemes and single-scale temporal modeling strategies. Early fusion approaches, which integrate features from different modalities at the input or representation level, can effectively exploit complementary cues and simplify model design. Late fusion methods, which combine predictions from modality-specific branches, offer modularity and robustness, allowing each modality to be optimized independently. Similarly, temporal modeling techniques based on single-scale architectures, such as LSTM, GRU, or vanilla Transformers, have been successful in capturing sequential patterns within individual modalities.

However, these static fusion and single-scale temporal methods have notable limitations. Static fusion assumes fixed modality contributions and uniform importance across all samples, potentially propagating redundant or uninformative signals and failing to account for varying modality reliability. Early fusion models, in particular, can struggle to separate salient from noisy modality-specific information, while late fusion approaches may miss deep cross-modal temporal interactions. Single-scale temporal models focus either on long-term trends or short-term fluctuations, but not both, limiting their ability to capture multi-scale temporal dynamics inherent in behavioral and affective signals. Furthermore, these approaches typically apply the same computation pipeline to all samples, resulting in unnecessary computational costs and potentially suboptimal performance in real-world multimodal depression analysis.

However, existing depression detection models face significant limitations in addressing these challenges. Many models overly rely on global pattern analysis and neglect the short-term fluctuations in emotions and behaviors, which restricts their ability to capture the dynamic changes in depression symptoms [24,25]. Furthermore, current models often fail to effectively integrate long-term and short-term information, making it difficult to accurately capture the subtle variations in depression from complex time-series data. Additionally, many models do not fully utilize multi-scale information, making it challenging to balance global long-term patterns with local short-term changes, resulting in detection outcomes that are neither comprehensive nor accurate.

In terms of multimodal fusion, although traditional multimodal fusion methods have made some progress, they typically rely on static fusion strategies that cannot dynamically adjust according to the characteristics of the input data [26,27,28]. This static approach often faces computational efficiency bottlenecks when dealing with large-scale data, particularly when there is redundant or insufficient information in the modal data. Static fusion methods cannot flexibly adapt between different tasks and input characteristics, leading to wasted computational resources and suboptimal performance. This limitation is especially apparent in scenarios with strict computational resource requirements, such as real-time processing and edge computing.

**Figure 1:**
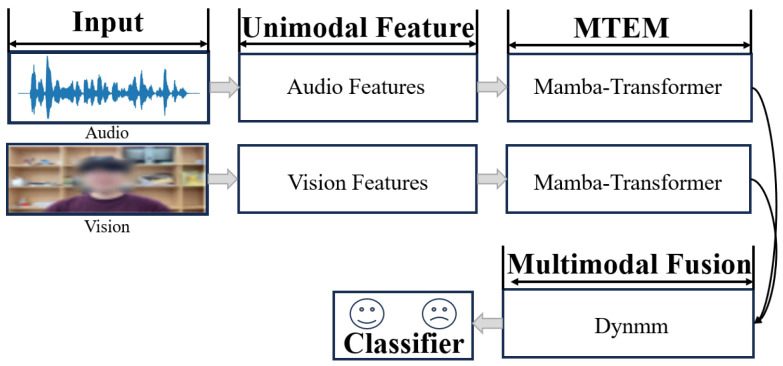
Motivation of Our Method. To capture temporal patterns, audio and visual features are independently processed by the MITEM module, in which the Mamba-Transformer enables parallel modeling of both short- and long-term dependencies. The DynMM module performs efficient multimodal fusion via dynamic path selection, leading to the final classification output.

Figure 1 illustrates the motivation behind our approach. The main contributions of this paper are as follows:

1. We propose a dynamic multimodal depression detection framework—DynMultiDep, which combines multi-scale temporal modeling and an adaptive fusion mechanism. The core innovation lies in the Multi-scale Temporal Expert Module (MTEM) and the Dynamic Multimodal Fusion Module (DynMM). MTEM extracts long-term trend features through Mamba experts and captures short-term fluctuations using a local window Transformer, achieving adaptive fusion through a long-short routing mechanism.
2. For the first time, we introduce the novel DynMM framework, a dynamic fusion approach, into the field of multimodal depression detection. It dynamically adjusts modality selection and fusion strategies based on the input features during both training and inference stages, significantly improving computational efficiency and adaptability. This method overcomes the limitations of traditional static fusion strategies and demonstrates higher efficiency when dealing with complex multimodal data.
3. DynMultiDep is the first multimodal depression detection framework to combine dynamic fusion and multi-scale temporal modeling, providing an effective solution to accurately capture subtle changes in depressive symptoms. Extensive experiments on two large depression datasets, D-Vlog and LMVD, show that our proposed method significantly outperforms existing state-of-the-art approaches.

The rest of the paper is organized as follows. Section 2 presents the related work on multimodal depression detection. Section 3 introduces the DynMultiDep framework. The experimental setup and results analysis are described in Section 4 and Section 5, respectively.

## 2. Related Work

### 2.1. Depression Detection Techniques

Traditional depression detection methods primarily rely on clinical assessments and psychological scales, such as the Hamilton Depression Rating Scale (HDRS) and the Self-Rating Depression Scale (SDS). However, these methods are highly subjective, inefficient, and time-consuming [29,30,31,32,33,34,35,36]. In recent years, data-driven depression detection methods have gradually become a research focus. These methods analyze multimodal data (e.g., speech, text, physiological, and visual signals) to comprehensively capture the features of depression. For instance, speech signal analysis can extract features such as pitch and speech rate to identify emotional fluctuations; text-based methods analyze language expressions, vocabulary usage, etc., to detect depressive tendencies; physiological signals (e.g., heart rate, skin conductance) and visual signals (e.g., facial expression analysis) are also widely applied for early detection [37,38,39]. Although unimodal methods are effective in certain scenarios, they are limited by the fact that they only capture a partial set of depression-related features, resulting in significant limitations.

### 2.2. Multimodal Fusion and Time Series Modeling

Multimodal data fusion is a core challenge in depression detection. Existing methods typically adopt static fusion strategies, where data from different modalities are simply concatenated and modeled together. However, this approach cannot dynamically adjust the modality weights based on input data, leading to poor adaptability and reduced detection performance. To address this, dynamic fusion methods have gained significant attention in recent years. These methods introduce attention mechanisms or path selection strategies to dynamically adjust the contribution of each modality, enhancing the flexibility and effectiveness of the models [40,41].

In time-series modeling, the Transformer architecture utilizes self-attention mechanisms to capture long-term and short-term dependencies, performing excellently. However, it still faces limitations in handling multi-scale features, particularly when dealing with data across different time scales [42]. To overcome this issue, recent studies have proposed improvements to the multi-scale Transformer approach, incorporating hierarchical attention mechanisms and temporal window techniques to enhance the model’s ability to handle multi-scale feature modeling. Therefore, the combination of dynamic multimodal fusion and multi-scale time-series modeling has become a key direction for improving the accuracy and adaptability of depression detection [43,44,45,46,47,48,49,50,51,52,53,54,55,56,57,58,59,60,61,62,63,64].

## 3. Methodology

**Figure 2:**
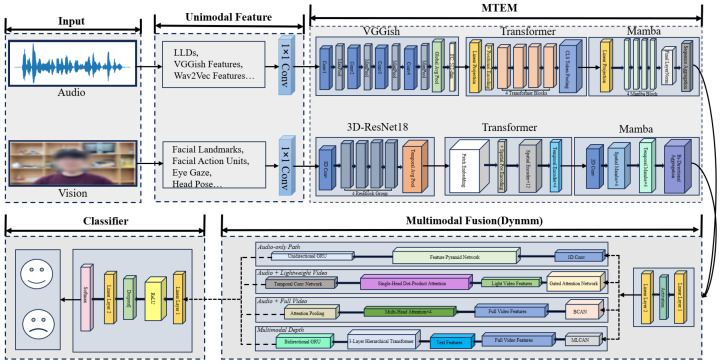
Figure 1 shows the overall framework of the proposed method, DynMultiDep. After the raw multi-modal signals are input, the feature extraction module extracts deep representations and predefined features. The features are then fed into the MT Expert Module, where the Mamba expert model captures long-term trend features, and the local window Transformer captures short-term dynamic fluctuations. These two components are dynamically fused through a long-short routing mechanism. The fused features are then input into the Dynamic Multimodal Fusion module, which dynamically adjusts modality selection and fusion strategies. Finally, the output is used for the depression classification task.

The proposed DynMultiDep framework consists of three core modules: multimodal feature extraction, multi-scale time series modeling (Multi-scale Temporal Expert Module, MTEM), and dynamic multimodal fusion (Dynamic Multimodal Fusion, DynMM). In the overall architecture, the feature extraction module first captures deep features and pre-designed features from the audio and visual modalities. Then, the MTEM module performs multi-scale temporal modeling on each modality to capture long-term global patterns and short-term local variations. Finally, the DynMM module adaptively fuses the information from different modalities to achieve efficient depression classification. The entire DynMultiDep framework is shown in Figure 2.

### 3.1. Multimodal Feature Extraction

This paper uses two modalities, audio and visual, to extract the corresponding features of each modality, leveraging deep learning models pre-trained on large-scale datasets for deep representation learning.

Acoustic Features: We use OpenSMILE to extract 25 low-level descriptors (LLDs) from audio, which effectively capture emotional and speech characteristics in the audio signal. Specifically, the low-level descriptors extracted by OpenSMILE include features related to frequency, loudness, pitch, speech rate, and more, such as Mel-frequency cepstral coefficients, pitch, energy, formants, and spectral envelope. These low-level features provide the foundational data for tasks like emotion analysis and speech understanding, helping us better comprehend and analyze audio signals.

For the audio modality, we first extract frame-level deep features using a pre-trained VGGish network, producing 128-dimensional embeddings per frame. To obtain a fixed-length representation for each audio segment, we aggregate these frame-level embeddings using three statistics: maximum, sum, and mean, resulting in three separate 128-D vectors that are concatenated into a final representation. This aggregation is performed over sliding windows with a fixed frame length and stride, allowing effective capture of temporal dynamics while suppressing noise. Additionally, we normalize loudness across frames to reduce inter-sample variability. Compared with segment-level temporal pooling, this global statistical aggregation captures overall signal characteristics more robustly, demonstrating superior performance in preliminary evaluations.

For visual features, we employ the dlib library (version X.X) for face detection and facial landmark extraction, followed by Action Unit (AU) analysis using OpenFace (version X.X) to quantify fine-grained facial expressions. For each frame, we extract AU activations, gaze direction, head pose, and eye movement features. Frames with failed face tracking are discarded, and linear interpolation is applied to maintain temporal continuity. This preprocessing ensures that subtle facial dynamics relevant to depression detection are preserved while mitigating the impact of occasional tracking failures.

Visual Features: In the emotion analysis task for depression detection, changes in facial expressions can provide key behavioral clues. We first use the pre-trained face detection model from dlib to locate the face region, then use the 68-point detector provided by dlib to obtain the facial coordinates for each frame. These coordinates reflect the dynamic facial expressions of the individual during the video recording, such as the curvature of the mouth, the degree of eyebrow raising, and the openness or closure of the eyes. Additionally, we extract other key behavioral features, including action units (AUs), gaze, head posture, and eye movement. AUs are based on facial muscle movements and can capture subtle changes in an individual’s expression. Research has shown that the AU activation patterns of individuals with depression often differ from those of non-depressed individuals. Gaze and eye movement information can reflect an individual’s visual attention patterns. Depressed individuals tend to have reduced eye contact in social interactions and often exhibit wandering eyes or long periods of avoiding specific targets. Head posture provides supplementary information about the individual’s overall behavior. Depressed individuals may engage in fewer active head movements, tending to lower their heads or avoid direct interaction with others. Previous studies have shown that individuals with depression typically have fewer facial expressions, and the duration of specific expressions may be longer. Therefore, these fine-grained geometric features can serve as important discriminative information.

In summary, this paper combines audio and visual modality features, extracting both low-dimensional predefined features and deep learning representations to enhance the discriminative ability for depression detection. For the features of each modality, we use 1D convolution to map them into the same dimensional space, preparing for effective fusion and processing of features from different modalities in the subsequent stages. Compared to single-modal methods, multimodal features can more comprehensively characterize the behavioral patterns of individuals with depression.

### 3.2. Multiscale Time Series Modeling (MTEM)

#### 3.2.1. Dynamic Multimodal Fusion and Signal Preprocessing

**Figure 3:**
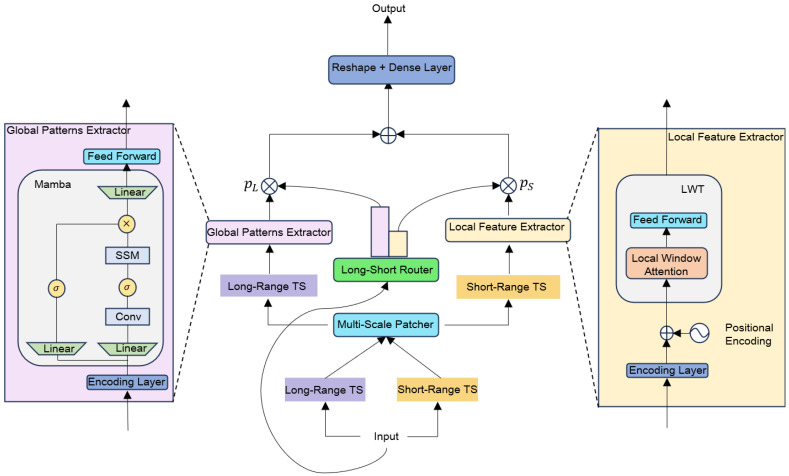
MTEM Overview: The multiscale patch module converts the input time series (TS) into different resolutions based on distance. Mamba serves as the low-resolution global expert, while LWT acts as the high-resolution local expert. The long-short router adaptively learns the contributions of both experts.

The extracted audio and visual features are input into a multimodal time series $x\in R^{L\times M}$ (where *L* is the sequence length and *M* is the number of modalities). These features are preprocessed through a multi-scale patch module to separate long-term trends from short-term dynamic features. First, the original sequence is split by modality into univariate sequences $\left\{X^{\left(1\right)},X^{\left(2\right)},\ldots ,X^{\left(M\right)}\right\}$. The sequence $x^{\left(i\right)}\in R^{L}$ is divided into low-resolution patches $X_{PL}^{\left(i\right)}\in R^{N_{L}\times P_{L}}$, where $N_{L}=\left\lfloor \frac{L-P_{L}}{Str_{L}}\right\rfloor +1$. This process aggregates time steps to suppress noise and retain global patterns (as shown in Figure 3). For capturing short-term dynamics, a smaller patch length $P_{L}$ and stride $Str_{L}$ are used to generate high-resolution patches $X_{PS}^{\left(i\right)}\in R^{N_{S}\times P_{S}}$, preserving fine-grained fluctuation information. After patching, the information content of the patched sequences is quantified by the PTS resolution defined in Equation (1):

(1) $$R_{PTS}=\frac{\sqrt{P}}{Str}$$

The long-range patch resolution $R_{PTS}=\frac{\sqrt{P_{L}}}{Str_{L}}$ is lower, focusing on global pattern modeling, while the short-range patch resolution $R_{PTS}=\frac{\sqrt{P_{S}}}{Str_{S}}$ is higher, enhancing local dynamic capture capabilities.

#### 3.2.2. The Signal Processing Flow of the Expert Module

The patched subsequences are fed into two types of expert modules for feature extraction:

##### Mamba Long-Term Trend Expert

Algorithm 1 presents the pseudocode implementation of the Mamba long-term trend expert algorithm, clearly illustrating its core logic and computational flow. The Mamba long-term trend expert takes low-resolution patches $X_{PL}^{\left(i\right)}\in R^{N_{L}\times P_{L}}$ as input signals. It first maps the input patches to a higher-dimensional latent space $x_{L}^{\left(i\right)}\in R^{N_{L}\times D}$ using a linear projection layer. This latent representation then enters the core Mamba block. Based on the state space model (SSM) paradigm, Mamba dynamically models long-term dependencies by adapting the foundational recurrence relation:

(2) $$h_{t}=\bar{A}h_{t-1}+\bar{B}x_{t},\;y_{t}=Cx_{t}$$

However, a key innovation in Mamba is its selectivity mechanism: the state transition matrix $\bar{A}$, input matrix $\bar{B}$, and output matrix *C* are not fixed learnable parameters but are functions of the input $x_{t}$, derived through learned transformations. This allows the model to selectively propagate or forget information based on the input context. The discretization process, involving a learnable timescale parameter Δ, transforms the continuous-time SSM parameters (A, B) into their discrete counterparts $\left(\bar{A},\bar{B}\right)$ used in Equation (2). Through this selective recurrence process, noise is effectively filtered, and cross-temporal global patterns are captured. For efficient training on parallel hardware, Mamba utilizes a parallel scan algorithm instead of direct sequential recurrence. The module ultimately outputs long-term feature encodings $z_{L}^{\left(i\right)}\in R^{N_{L}\times D}$, achieving linear time complexity O(L) with respect to sequence length, ensuring efficient modeling of long sequences.
**Algorithm 1 **MambaBlock**Require: **Feature sequence input_features of shape [batch_size, seq_len, d_model] **     Optional Parameters:** hidden_dim = 1024, state_dim = 16, conv_kernel = 4

**Ensure: **Output feature sequence of shape [batch_size, seq_len, d_model]
   1:Normalize input_features using **LayerNorm**   2:Project input_features to hidden_dim using **Linear**(x, d_model → hidden_dim)   3:Apply **1D Convolution** on projected features with kernel_size=conv_kernel   4:Apply Activation “silu” to convolution output   5:Generate state space parameters:   6:     Set **A** as a fixed state transition matrix (LinearB with shape [state_dim, state_dim])   7:     Calculate dynamic time step **dt** using **Linear**(x_proj, hidden_dim → hidden_dim)   8:     Apply **Softplus** to dt to ensure positivity   9:     Calculate **B** using **Linear**(x_proj, hidden_dim → state_dim) 10:     Calculate **C** using **Linear**(x_proj, hidden_dim → state_dim) 11:Perform **Selective Scan Algorithm** to process long-term dependencies: 12:     h[t]=A(Δ[t])·h[t−1]+B[t]·x[t] 13:     y[t]=C[t]·h[t] 14:Apply **mixing gate mechanism**: 15:     Calculate **gate** using **Linear**(x, d_model → hidden_dim) 16:     Apply Activation “silu” to gate 17:     Apply gate modulation: y=y∗gate 18:Project output y back to original dimension using **Linear**(y, hidden_dim → d_model) 19:Add **residual connection**: output = output + input_features 20:**Return** output

##### Local Window Transformer (LWT) Short-Term Fluctuation Expert

Algorithm 2 presents the pseudocode implementation of the Local Window Transformer (LWT) short-term fluctuation expert, providing an intuitive illustration of the algorithm’s core logic and computational steps in capturing short-term fluctuations. The Local Window Transformer (LWT) receives high-resolution patches $X_{PS}^{\left(i\right)}\in R^{N_{S}\times P_{S}}$ as input. After adding learnable positional encodings to incorporate sequence order information, the input is projected to a D-dimensional representation $x_{S}^{\left(i\right)}\in R^{N_{S}\times D}$. A typical LWT layer, analogous to a standard Transformer encoder layer, consists of two main sub-layers: multi-head self-attention (MHSA) applied within local windows, and a position-wise feed-forward network (FFN). Residual connections and layer normalization are applied around each sub-layer to facilitate training.

The core of the LWT lies in its fixed-window multi-head self-attention mechanism. Instead of attending to the entire sequence, each position only attends to positions within a surrounding window of fixed size *w*. Within each attention head, the computation follows the standard scaled dot-product attention, as shown in Equation (3):

(3) $$\mathrm{Attention}\left(Q_{w},K_{w},V_{w}\right)=\mathrm{softmax}\left(\frac{Q_{w}K_{w}^{T}}{\sqrt{d_{k}}}\right)V_{w}$$

Here, $Q_{w},K_{w},V_{w}\in R^{w\times d_{k}}$ are the query, key, and value vectors derived from the input tokens within the current local window. $d_{k}$ is the dimension of keys/queries per head. The window size *w* strictly restricts the attention scope, forcing the model to focus on capturing local dynamics and contextual information within adjacent steps. Following the windowed MHSA, the position-wise feed-forward network (FFN), typically composed of two linear transformations with a non-linear activation in between, further processes the features independently at each position.

By stacking multiple layers (*l*) of LWT, the model gradually expands its effective receptive field to approximately l×w, allowing for interactions across windows while still preserving fine-grained fluctuation features captured by the local attention mechanism. The final output comprises short-term feature encodings $z_{S}^{\left(i\right)}\in R^{N_{S}\times D}$. Crucially, the computational complexity per layer is significantly reduced from the $O(N_{S}^{2}D)$ of a standard Transformer to approximately $O(N_{S}w^{2}D)$ (or potentially $O(N_{S}wD)$ depending on implementation details), making it linear in sequence length $N_{S}$ for a fixed window size *w*. This efficiency meets the processing requirements for high-resolution sequences where capturing short-term dynamics is paramount.
**Algorithm 2 **TransformerBlock**Require: **Feature sequence input_features of shape [batch_size, seq_len, d_model] **     Optional Parameters: ** heads = 8, head_dim = 64, ff_dim = 2048

**Ensure: **Output feature sequence of shape [batch_size, seq_len, d_model]
  1:Normalize input_features using **LayerNorm**  2:Perform **Multi-Head Self-Attention**:  3:     Generate query q, key k, and value v using **Linear**(x, **d_model**→ **heads** * **head_dim**)  4:     Reshape q,k,v to multi-head format: [batch_size, seq_len, heads, head_dim]  5:     Calculate attention scores as **MatMul**(q, **Transpose**(k, [0, 1, 3, 2])) / sqrt(**head_dim**)  6:     If mask is provided, apply mask to scores: scores=scores+mask (−1e9)  7:     Calculate attention weights using **Softmax**(scores, axis = −1)  8:     Compute context by weighted sum: context=MatMul(weights,v)  9:     Reshape context to [batch_size, seq_len, heads * head_dim]10:     Project context to output using **Linear**(context, heads * head_dim → **d_model**)11:     Apply first **residual connection**: x = **input_features** + **attn_output**12:Normalize **x** using **LayerNorm**13:Perform **Feed-Forward Network**:14:     Apply **Linear**(x, **d_model** → **ff_dim**)15:     Apply **GELU** activation to **ff_output**16:     Project **ff_output** back to **d_model** using **Linear**(ff_output, **ff_dim** → **d_model**)17:Apply second **residual connection**: **output** = **x** + **ff_output**18:**Return** output

### 3.3. Dynamic Feature Fusion

To integrate information from different temporal scales, the long-term features ($z_{L}^{\left(i\right)}$) and short-term features ($z_{S}^{\left(i\right)}$) are adaptively fused via a dynamic long-short routing mechanism. This mechanism weights the contribution of each expert based on the global characteristics of the input.

First, a global context vector $z_{B}\in R^{D}$ is derived from the original uni-modal input sequence $X^{\left(i\right)}$, for instance, through global average pooling followed by a linear transformation. This vector $z_{B}$ summarizes the overall nature of the sequence.

Next, as shown in Equation (4), $z_{B}$ is passed through a gating network (a linear layer and Softmax function) to generate dynamic expert weights $p_{L}$ and $p_{S}$:

(4) $$\left[p_{L},p_{S}\right]=\mathrm{softmax}\left(Wz_{B}+b\right)$$

where $p_{L}+p_{S}=1$. These weights reflect the judged importance of long-term versus short-term representations for the given input.

Finally, as shown in Equation (5), the features are fused by scaling each sequence by its weight and then concatenating them along the sequence dimension:

(5) $$z_{LS}^{\left(i\right)}=\mathrm{concat}\left(p_{L}\cdot z_{L}^{\left(i\right)},\;p_{S}\cdot z_{S}^{\left(i\right)};\;\dim=\mathrm{sequence}\right)$$

The resulting fused features $z_{LS}^{\left(i\right)}\in R^{(N_{L}+N_{S})\times D}$ combine both long-term context and fine-grained details, adaptively emphasizing the more relevant temporal scale based on input characteristics. This enhances the robustness of the representation for downstream tasks.

### 3.4. Dynamic Multimodal Fusion (DynMM)

**Figure 4:**
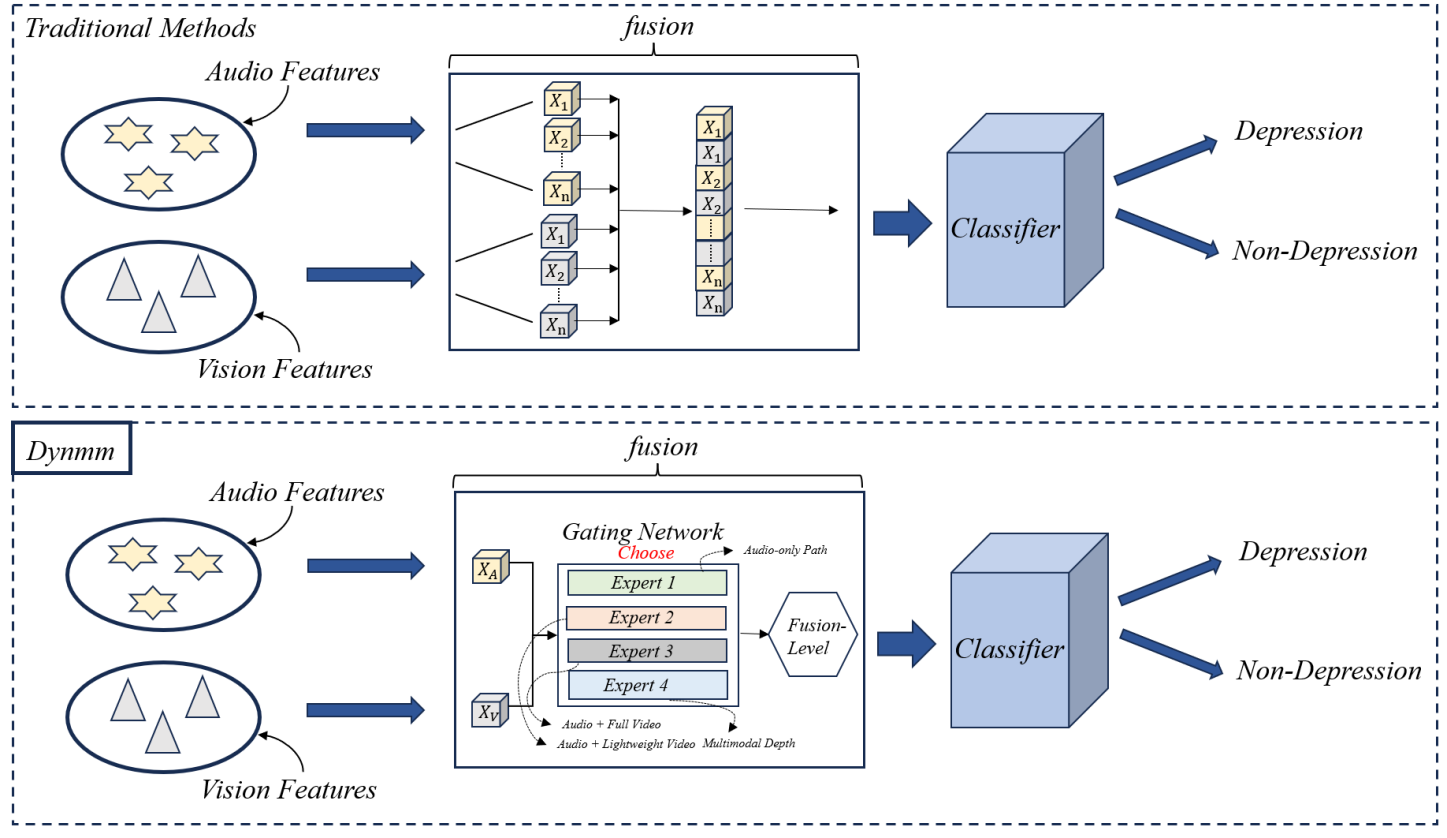
Comparison of Traditional Modality Fusion and DynMM: Schematic Overview.

Figure 4 compares the schematic diagrams of traditional modality fusion methods and DynMM, clearly presenting the different strategies employed by each in handling multimodal information. Traditional modality fusion methods typically rely on static fusion strategies, which simply combine the information from different modalities, making them ineffective in dealing with issues such as modality loss or data disturbances. In contrast, DynMM introduces a dynamic modeling mechanism, which adjusts the inter-dependency of modalities in real-time, effectively enhancing the model’s robustness when faced with incomplete or unstable data. Next, we will provide a detailed explanation of the specific working principles of DynMM, including how it optimizes the fusion process of multimodal data through dynamic weight allocation and modality recovery strategies, ensuring the stability and accuracy of the model in various environments.

### 3.5. Modality-Level Dynamic Decision Making

To provide a tiered computational framework ranging from lightweight unimodal probes to deep cross-modal analysis, we specifically design four expert networks ($E_{1}$ to $E_{4}$). This number of experts reflects a logical progression of multimodal integration complexity, allowing the gating network to prioritize computational efficiency for clear-cut samples while reserving deep fusion for ambiguous cases.

**Figure 5:**
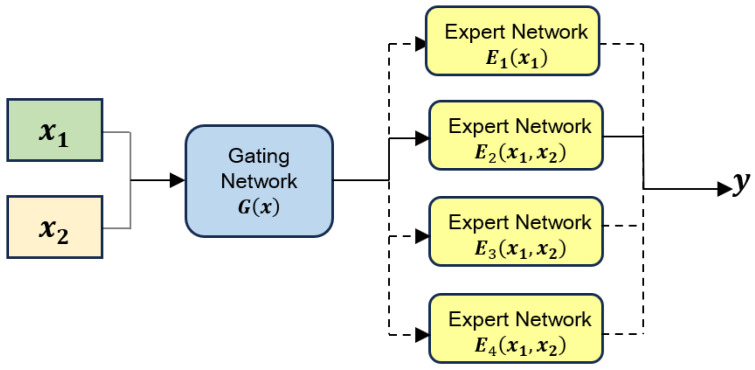
In the diagram of modality-level DynMM, the input data consists of two modalities and their representations, with the output being the result. The model contains multiple expert networks, each responsible for handling different modality combinations. The gating network G(x) makes dynamic decisions based on the input data, selecting the most appropriate expert network for processing.

Assume the primary input consists of features derived from two modalities: Audio (a) and Video (v), represented by feature vectors $x_{a}$ and $x_{v}$ respectively. To dynamically select the most appropriate processing strategy based on input characteristics, this approach employs a set of four specialized expert networks, $\left\{E_{1},E_{2},E_{3},E_{4}\right\}$, each tailored to a different level of multimodal analysis and computational load. Figure 5 provides a conceptual illustration of this modality-level dynamic architecture, showing how input modalities are processed by different expert networks under the control of a gating network. Each expert network is specialized as follows:

- $E_{1}\left(X_{1}\right)$ where $X_{1}=\left\{x_{a}\right\}$: Represents the Audio-Only processing pathway. This expert analyzes information solely from the audio modality $x_{a}$. It incurs the lowest computational cost (approx. 0.6G FLOPs).
- $E_{2}\left(X_{2}\right)$ where $X_{2}=\left\{x_{a},x_{v}\right\}$: Represents the Audio + Lightweight Video pathway. This expert first applies compressed sensing techniques to the input video features $x_{v}$ to sample and reconstruct a reduced representation, $x_{v\_cs}$, preserving key information while reducing data volume. It then processes the audio features $x_{a}$ alongside these lightweight video features $x_{v\_cs}$. This pathway offers a balance between incorporating basic audio-visual cues and maintaining high computational efficiency (approx. 1.0 G FLOPs).
- $E_{3}\left(X_{3}\right)$ where $X_{3}=\left\{x_{a},x_{v}\right\}$: Represents the Audio + Standard Video pathway. This expert processes the original audio features $x_{a}$ and the original video features $x_{v}$ directly, enabling a standard level of audio-visual analysis using the full video information. It operates at a moderate complexity level (approx. 1.6 G FLOPs).
- $E_{4}\left(X_{4}\right)$ where $X_{4}=\left\{x_{a},x_{v}\right\}$: Represents the Deep Audio-Visual Fusion pathway. This expert also processes the original audio $x_{a}$ and video $x_{v}$ features but employs the most sophisticated analysis techniques for deep multimodal integration. It entails the highest computational cost (approx. 2.3 G FLOPs) and is designated for the most challenging samples.

A modality-level gating network $G_{m}$ determines which single expert pathway ($E_{1}$ to $E_{4}$) is most appropriate for the current input. Based on the original input features $x_{a}$ and $x_{v}$, $G_{m}$ computes a score for each pathway according to the project’s specific design. Let these scores be $\mathrm{scores}\in R^{4}$. A sparse 4-dimensional decision vector $g\in {\left\{0,1\right\}}^{4}$ is generated via an argmax selection followed by one-hot encoding(see Equation (6)):

(6) $$g=\mathrm{one-hot}\left(\mathrm{argmax}(\mathrm{scores})\right)$$

This vector *g* activates exactly one expert. The final output $y_{m}$ is the result from this single selected expert(see Equation (7)):

(7) $$y_{m}=\sum_{k=1}^{4}g_{k}E_{k}\left(X_{k}\right)=E_{\mathrm{selected}}\left(X_{\mathrm{selected}}\right)$$

where $X_{k}$ is the specific set of input features required by expert $E_{k}$ (note that for $E_{2}$, $X_{2}=\left\{x_{a},x_{v}\right\}$ implies it receives both but internally processes $x_{v}$ into $x_{v\_cs}$).

This dynamic expert selection mechanism routes each input to one of the four pathways, adapting the analysis complexity to the sample. This optimizes performance while achieving significant computational savings by executing only the single selected expert network. Algorithm 3 presents the pseudocode implementation detailing this gated expert selection algorithm.
**Algorithm 3 **Dynamic Path Selection in DynMM**Require: **Initial audio features $\mathrm{fa}\in R^{d}$ **Ensure: **Selected path index p∈{1,2,3,4}  1:Extract initial audio features using **LightAudioEncoder**:  2:     fa_light=LightAudioEncoder(audio_input)  3:Perform **Path Gating Network** for complexity estimation:  4:     x=Linear(fa_light,512→256)// First linear projection  5:     x=ReLU(x)// Activation function  6:     x=Dropout(x,p=0.2)// Regularization  7:     x=Linear(x,256→4)// Second linear projection to path scores  8:     path_scores=Softmax(x)// Convert to probability distribution  9:Calculate **confidence score**:10:     conf_score=max(path_scores)// Maximum probability as confidence11:Perform **Path Selection** based on thresholds:12:     **if** $\mathrm{conf}\_\mathrm{score}>\tau_{1}\;\left(0.75\right)$ **then**13:         p=argmax(path_scores)// Select highest probability path14:         **if** p==1 **then**15:            **return 1**                   // Audio-only path16:         **else**17:            **if** conf_score>0.9 **then**18:                **return** p             // Use predicted path19:            **else**20:                **return** min(p+1,4) // Use more complex path for borderline cases21:     **else**22:         **if** path_scores[1]>0.4 **then**23:            **return 2**                   // Default to audio+light video24:         **else**25:            **return 4**                   // Default to full modality for uncertain cases26:**Return** p

#### 3.5.1. Fusion-Level Dynamic Decision Making

**Figure 6:**
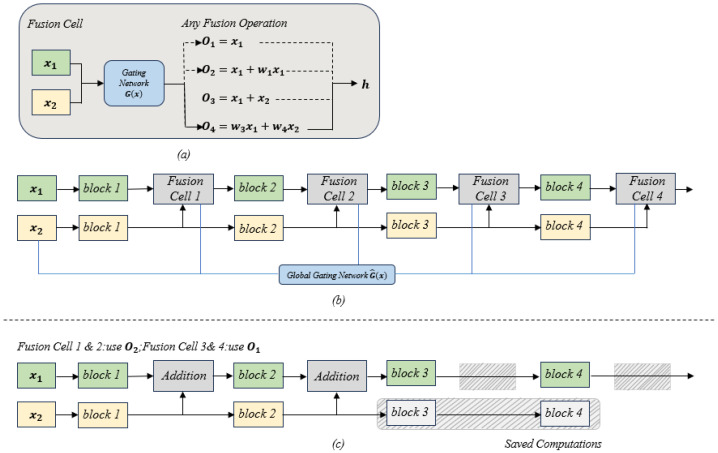
(**a**) Example architecture of the fusion-level DynMM, with inputs from two modalities $x_{1}$ and $x_{2}$. The fusion unit consists of a set of candidate operations $\left\{O_{i}\right\}$ (where $O_{1}$–$O_{4}$ denote Audio-Internal, Lightweight A-V, Standard A-V, and Deep A-V fusion, respectively) and a gating network G(x), where *H* is the output. (**b**) Dynamic multimodal architecture, where fusion units and static feature extraction blocks are alternately stacked. The gating networks G(x) of the four fusion units are integrated into a global gating network G(x), which outputs decisions for all four units at once. (**c**) An example architecture when the gating network selects $O_{2}$ for the first two fusion units and $O_{1}$ for the last two. This design saves computation for $x_{2}$ in fusion units 3 and 4 and their corresponding feature extraction units 3 and 4.

To dynamically refine the feature interaction strategy, DynMM introduces fusion units (Fusion-Cells). Each Fusion-Cell dynamically selects a fusion operation from a set of candidates $\left\{O_{1},O_{2},\ldots ,O_{F}\right\}$, embodying different fusion strategies (F=4). Figure 6a illustrates the concept.

Each fusion unit contains a set of candidate operations $\left\{O_{1},O_{2},\ldots ,O_{F}\right\}$. In our design, these operations correspond to different levels of fusion complexity, mirroring the strategies of the four processing pathways (F=4):

- $O_{1}\left(X\right)$ (Audio-Internal Fusion—Path 1 Strategy): This operation focuses on refining the uni-modal audio representation. It performs internal audio feature fusion by integrating representations from different levels of the audio encoder. It employs a feature pyramid structure or utilizes skip connections to combine shallow acoustic features with deep semantic features. Furthermore, it applies multi-scale aggregation with feature weighting mechanisms to adaptively emphasize relevant temporal scales or feature levels within the audio modality. This operation involves no cross-modal fusion.
- $O_{2}\left(X\right)$ (Lightweight Audio-Visual Fusion—Path 2 Strategy): This operation performs lightweight cross-modal fusion between audio ($x_{a}$) and lightweight video features ($x_{v\_light}$ or $x_{v\_cs}$). Its architecture prioritizes efficiency through a selective gating mechanism, where audio features generate signals controlling the activation of relevant lightweight video features. It utilizes a simplified, single-head attention mechanism for basic interaction, focusing on shallow feature interaction. Dimensionality reduction (e.g., 512→128) is applied to features before interaction to reduce computational load. The goal is to capture fundamental cross-modal cues efficiently.
- $O_{3}\left(X\right)$ (Standard Audio-Visual Fusion—Path 3 Strategy): This operation executes standard, comprehensive cross-modal fusion between audio ($x_{a}$) and full video features ($x_{v}$). It relies on bidirectional multi-head cross-attention (e.g., using 4 attention heads) for rich interaction (A→V and V→A). Fusion occurs at intermediate feature levels, and residual connections preserve the original modality information while incorporating cross-modal enhancements. This structure enables the learning of diverse cross-modal relationships.
- $O_{4}\left(X\right)$ (Deep Audio-Visual Fusion—Path 4 Strategy): This operation implements the most sophisticated deep multimodal fusion strategy for audio ($x_{a}$) and full video features ($x_{v}$). It employs a multi-level interaction framework (e.g., a 3-layer structure) for progressive, deep fusion. It utilizes co-attention mechanisms, incorporates fine-grained temporal alignment techniques between audio and video frames, and applies hierarchical feature fusion combining features from shallow to deep layers. The architecture integrates these deeply fused features, potentially using self-attention in a joint space, followed by a decoder structure (e.g., Transformer decoder) to interpret the representation for the final task. This operation aims to capture the most complex inter-modal dependencies.

The fusion-level gating network $G_{f}\left(X\right)$, associated with each fusion unit (or potentially globally shared as in Figure 6b), dynamically selects which operation is most suitable at the current stage *j*. It generates a decision vector $g_{j}\in {\left\{0,1\right\}}^{F}$(see Equation (8)):

(8) $$h_{j}=\sum_{k=1}^{F}g_{j,k}O_{k}\left(X\right),\;\mathrm{where}\;g_{j}=\mathrm{one-hot}\left(\mathrm{argmax}(G_{f}\left(X\right))\right)$$

Here, $h_{j}$ is the output of the *j*-th fusion unit, resulting from the single selected fusion operation $O_{k}$ for which $g_{j,k}=1$.

By stacking multiple fusion units (*N* units), DynMM achieves progressive fusion. Early units might preferentially select simpler operations (e.g., $O_{1}$ or $O_{2}$). Conversely, later units can dynamically invoke more complex operations (e.g., $O_{3}$ or $O_{4}$) if the gating network determines deeper multimodal reasoning is necessary. For instance, for samples where audio features are dominant, the dynamic mechanism might consistently select $O_{1}$ or $O_{2}$ in later units. This avoids the significant computational cost associated with executing complex operations like $O_{3}$ or $O_{4}$, as illustrated by the grayed-out modules conceptualized in Figure 6c.

#### 3.5.2. End-to-End Training and Classification Process

To balance classification accuracy and computational efficiency, DynMM’s training objective combines the task loss ($L_{\mathrm{task}}$, e.g., cross-entropy) with constraints on the expected computational cost, formulated as(see Equation (9)):

(9) $$L=L_{\mathrm{task}}+\lambda \left(\sum_{i=1}^{B}p_{i}C\left(E_{i}\right)+\sum_{j=1}^{N}\sum_{k=1}^{F}p_{j,k}C\left(O_{k}\right)\right)$$

where $C(E_{i})$ and $C(O_{k})$ represent the estimated computational costs (e.g., FLOPs) of the *i*-th modality-level expert network (B=4) and the *k*-th fusion-level operation (F=4, corresponding to Path 1-4 complexities), respectively. $p_{i}$ and $p_{j,k}$ are the selection probabilities from the respective gating networks (before argmax/one-hot), and λ is a hyperparameter controlling the trade-off. Due to the inherent discreteness of the argmax/one-hot selection in the gating mechanisms, DynMM employs the Gumbel-Softmax reparameterization technique during training to obtain differentiable “soft” decision vectors (see Equation (10)):

(10) $$\tilde{g_{k}}=\frac{\exp(\left(\log\pi_{k}+b_{k}\right)/\tau )}{\sum_{l=1}^{K}\exp\left(\left(\log\pi_{l}+b_{l}\right)/\tau \right)},\;b_{k}∼\mathrm{Gumbel}\left(0,1\right)$$

Here, $\pi_{k}$ represents the pre-softmax logits output by a gating network for the *k*-th choice (out of K=B=4 or K=F=4 choices), τ is the temperature parameter annealed during training (from high to low), and $b_{k}$ are samples from a Gumbel distribution. Gradients are typically propagated through the straight-through estimator (STE) during backpropagation to maintain stable training.

After multimodal features are extracted, they are processed by the DynMM module. First, the modality-level gating $G_{m}$ selects the primary expert branch $E_{i}$. The features, potentially processed by $E_{i}$, are then passed through the stack of *N* fusion units. Within each unit *j*, the fusion-level gating $G_{f}$ selects the appropriate operation $O_{k}$ (reflecting Path 1–4 complexity), progressively fusing multimodal information. The final features $h_{N}$ from the last fusion unit are mapped to classification probabilities via a fully connected layer and Softmax:

(11) $$p_{class}=\mathrm{Softmax}\left(W_{final}h_{N}+b_{final}\right)$$

The entire model, including feature extractors (if applicable within the experts/ operations), gating networks, and expert/fusion operations, is trained end-to-end using the combined loss objective (Equation (11)). This allows the model to learn not only the classification task but also how to dynamically adapt its internal structure based on the multimodal input data, thereby achieving both efficient and accurate classification.

## 4. Experimental Setup

### 4.1. Datasets and Baselines

To validate the generalizability and effectiveness of our model, we conducted experiments on two large-scale depression datasets: the D-Vlog dataset [65] and the LMVD dataset [66].

1. D-Vlog: D-Vlog is a multimodal depression dataset focused on analyzing the non-verbal behaviors of individuals with depression. The dataset consists of 961 video logs, with 555 videos depicting individuals with depression and 406 videos representing non-depressed individuals. It includes visual, audio, and text features, capturing multidimensional characteristics of depression symptoms.
2. LMVD: LMVD is a large-scale multimodal depression dataset specifically designed for audiovisual depression detection, with data sourced from social media platforms. The dataset includes visual, audio, and text data, providing rich emotional expression information to help identify depression-related features.
3. Baselines: First, on the D-Vlog dataset, we employ four state-of-the-art methods (MCRVT [67], Spike Memory Transformer [68], JAMFN [69], and DepMamba [70]) as baselines. Second, on the LMVD dataset, we use two state-of-the-art methods (DepMamba and LMTformer [71]) as baselines, with a focus on designing multimodal (video and audio) methods.

For both datasets, data preprocessing involved voice activity detection (VAD) to remove silent segments and Min-Max normalization of visual landmarks. Samples were excluded based on strict criteria: (i) videos where face tracking confidence (via dlib) fell below 0.8 for more than 20% of the frames, and (ii) audio recordings with a signal-to-noise ratio (SNR) below 15dB. The final cohorts represent a diverse demographic of social media vloggers, with ages typically ranging from 18 to 45, ensuring broad applicability of the non-verbal behavioral patterns captured.

Additionally, on both the D-Vlog and LMVD datasets, we compare seven baseline methods: Transformer [72], LSTM [73], BiLSTM [74], GRU [75], TCN [76], GNN [77], and MMDNet [78], with designs considering both unimodal (audio, video) and multimodal (audio and video) perspectives. To ensure experimental fairness, all models are evaluated under the same parameter settings and experimental environment.

### 4.2. Implementation Details

All experiments were conducted on a 64-bit Windows 10 system equipped with an NVIDIA RTX 4090 GPU.

1. Optimizer and Training Settings: The Adam optimizer was used with a learning rate of 1e-4 and a batch size of 16. To ensure reproducibility, a fixed random seed of 42 was applied to all experimental runs. Hyperparameter tuning was conducted using a grid search strategy on the validation set. Training was managed via an early stopping mechanism, terminating if the validation loss failed to improve for 15 consecutive epochs, within a maximum limit of 120 epochs.
2. Feature Extraction: For the video and audio modalities, the Mamba model (state dimension = 10) and the Transformer model (hidden dimension = 256) were employed. The data was processed through a 1×1 convolution layer (256 channels), followed by Mamba and Transformer blocks to extract long-term dependencies and local features.
3. Multimodal Fusion: A gating network (hidden dimension = 256) was designed to decide whether to activate the single-modality path or fuse the multimodal path. A resource-aware loss function was introduced to balance the task loss and computational cost.
4. Dataset Splitting: The D-Vlog dataset was split into training, validation, and test sets in a 7:1:2 ratio, while the LMVD dataset was split in an 8:1:1 ratio. For both D-Vlog and LMVD datasets, we ensured strict subject-level separation across training, validation, and testing sets. Specifically, no individual appears in more than one subset, preventing potential identity leakage that could artificially inflate performance metrics.
5. Evaluation Metrics: Six evaluation metrics were employed, including accuracy, precision, recall, F1-score, unweighted accuracy (UA), and weighted average F1 (WF1) [79].

## 5. Results and Analysis

The superior performance of the DynMultiDep model has been validated through extensive experiments, and the underlying reasons for its effectiveness have been thoroughly explored. The following research questions (RQs) are addressed based on the experimental results:

- RQ1: Does DynMultiDep demonstrate strong generalization ability when applied to different datasets?
- RQ2: In what aspects does the DynMultiDep model outperform existing state-of-the-art methods?
- RQ3: How does DynMultiDep effectively improve detection accuracy through dynamic multimodal fusion and multiscale time-series modeling?
- RQ4: Can DynMultiDep maintain stable detection performance under different noise levels?
- RQ5: How does DynMultiDep perform when handling missing or disrupted modalities?

### 5.1. Our Method vs. Baseline Models: A Comparative Study (RQ1)

Table 1 shows that in the multimodal task on the D-Vlog dataset, DynMultiDep achieves an accuracy of 0.8, significantly outperforming all baseline models, demonstrating its exceptional classification ability. Although models like LSTM have a higher recall rate in the audio task, their accuracy is only 0.5849, indicating a substantial deviation. In contrast, DynMultiDep, through its dynamic cross-modal interaction mechanism, not only achieves the highest accuracy but also maintains high precision and recall, improving overall performance. For the audio task, DynMultiDep achieves an accuracy of 0.7692, surpassing MMDNet, while in the visual task, it reaches an accuracy of 0.7745, which is 13.7% higher than TCN, demonstrating its advantage in dynamic visual pattern capture.

Table 2 shows that in the multimodal task on the LMVD dataset, DynMultiDep continues to maintain its leading position, with the highest accuracy, highlighting its robustness across different data distributions. The accuracy of the audio unimodal task is 0.66, surpassing GRU. Although the F1 score is slightly lower, the higher precision indicates better classification performance for the majority class samples. In the visual task, DynMultiDep leads with an accuracy of 0.7452, surpassing ResNet by 7.67%.

Cross-dataset analysis reveals that DynMultiDep improves the multimodal accuracy by 13.96% on the D-Vlog dataset and 3.83% on the LMVD dataset, validating its generalization ability. Despite the sample imbalance in LMVD, DynMultiDep maintains the highest accuracy through its dynamic feature selection mechanism, and it outperforms other models in balancing precision and recall, demonstrating its robust performance.

### 5.2. Comparison Between Our Method and SOTA Models (RQ2)

To ensure fairness in the experiments, comparative experiments were conducted under identical experimental settings. The results demonstrate that DynMultiDep significantly outperforms the existing state-of-the-art (SOTA) models in the depression detection task. Table 3 shows that, on the D-Vlog dataset, the performance of DynMultiDep surpasses that of models such as MCRVT (63.1%), Splice Memory Transformer (70.73%), JAMFN, and DepMamba (67.87%), particularly excelling in key metrics such as precision (79.17%), recall (89.06%), and F1 score (83.82%). These results validate its effectiveness in capturing both global and local features of depressive symptoms.

In Table 4, DynMultiDep also outperforms DepMamba (70.13%) and LMTformer (74.22%) on the LMVD dataset, achieving an accuracy of 76.51%, while maintaining leadership in precision (75.86%), recall (82.50%), and F1 score (79.04%). These results confirm the superiority of DynMultiDep’s dynamic fusion mechanism and multi-scale temporal modeling approach, particularly in its ability to adaptively integrate multimodal information while maintaining high computational efficiency.

The stable performance across datasets and tasks provides strong evidence of DynMultiDep’s reliability and generalization ability as an intelligent tool for depression detection.

### 5.3. Ablation Study (RQ3)

To validate the independent contributions and synergistic effects of the Multimodal Temporal Enhancement Module (MTEM) and the Dynamic Multimodal Fusion Module (DynMM) in depression detection, ablation experiments were conducted on the D-Vlog and LMVD datasets. The experiments included two groups: one where DynMM was removed, leaving only MTEM, and another where MTEM was removed, leaving only DynMM. By comparing the performance of the complete model with the ablation models, the roles of each module in multi-scale temporal modeling and dynamic fusion were analyzed.

#### 5.3.1. D-Vlog Dataset

**Figure 7:**
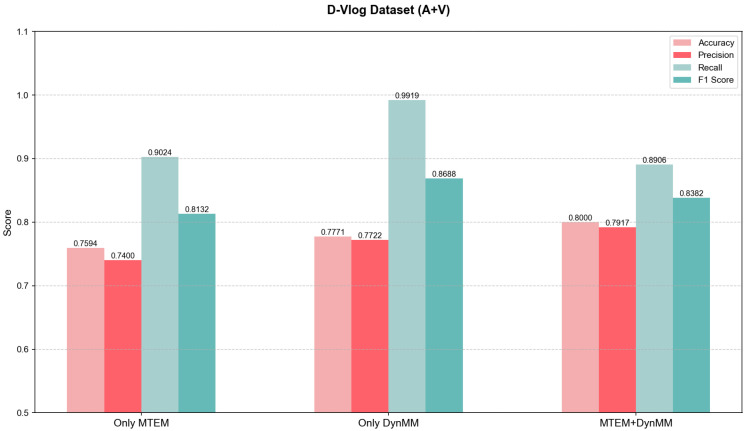
Ablation experiment performance comparison on the D-Vlog dataset.(Error bars denote SD; N=961 subjects).

As shown in Figure 7. When only MTEM was retained, the accuracy was 0.7594 and the F1 score was 0.8132, which were lower than the complete model (0.8000). The absence of DynMM’s dynamic interaction led to the inability of modal weights to adaptively adjust, and redundant visual features interfered with information alignment. When only DynMM was retained, the accuracy was 0.7771 and the F1 score was 0.8688, but due to the lack of temporal enhancement from MTEM, the model was affected by noise in the audio features. The complete model improved by 4.06% compared to MTEM and by 2.29% compared to DynMM, demonstrating the complementarity between the two modules.

#### 5.3.2. LMVD Dataset

**Figure 8:**
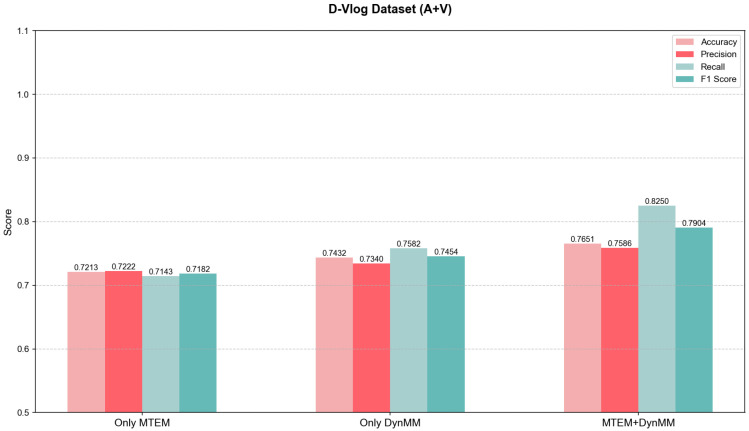
Ablation experiment performance comparison on the LMVD dataset(Error bars denote SD; N=254 subjects).

As shown in Figure 8. When only MTEM was used, the accuracy was 0.7213, lower than that of the complete model (0.7651). The absence of DynMM’s cross-modal interaction led to mismatched speech and facial expression information. When only DynMM was retained, the accuracy was 0.7432, 2.19% lower than the complete model, primarily due to insufficient temporal resolution. The complete model significantly improved performance through the temporal enhancement of MTEM and the dynamic fusion of DynMM.

#### 5.3.3. Synergistic Analysis

The experimental results indicate that the synergistic design of MTEM and DynMM significantly optimized the model’s performance. MTEM provides strong unimodal feature representation, while DynMM performs complementary fusion by dynamically selecting modalities. The combination of both effectively eliminates redundancy and enhances the model’s robustness, validating the deep coupling between temporal modeling and dynamic fusion.

### 5.4. Visual Analysis

#### 5.4.1. Statistical Significance Analysis

To validate the effectiveness of the proposed method on different datasets, it was compared with seven baseline methods, and statistical significance was analyzed using independent samples *t*-tests. The accuracy distributions of each method across 30 independent experiments were recorded, and the mean difference (Mean Diff), effect size (Cohen’s d), and adjusted *p*-values were calculated. To control for the effect of multiple comparisons, the Bonferroni correction was applied to adjust the significance level, ensuring the rigor of the analysis.

The experimental results on the D-Vlog dataset (see Table 5) show that the proposed method achieved statistically significant improvements over all baseline models (p<0.05). Compared to Transformer, LSTM, BiLSTM, GRU, TCN, ResNet, and MMDNet, the mean differences were 0.151820, 0.252626, 0.165838, 0.236973, 0.194799, 0.234569, and 0.164750, respectively. The Cohen’s d values for all comparisons were greater than 7, indicating that the proposed method significantly outperforms the baseline models on this dataset.

The statistical analysis on the LMVD dataset (see Table 6) further confirmed the superiority of the proposed method. The mean differences ranged from 0.061750 to 0.110838, with Cohen’s d values exceeding 3, and all *p*-values were less than 0.05, demonstrating the robustness of the method on this dataset.

#### 5.4.2. Cross-Time Attention Patterns in Multimodal Depression Detection

**Figure 9:**
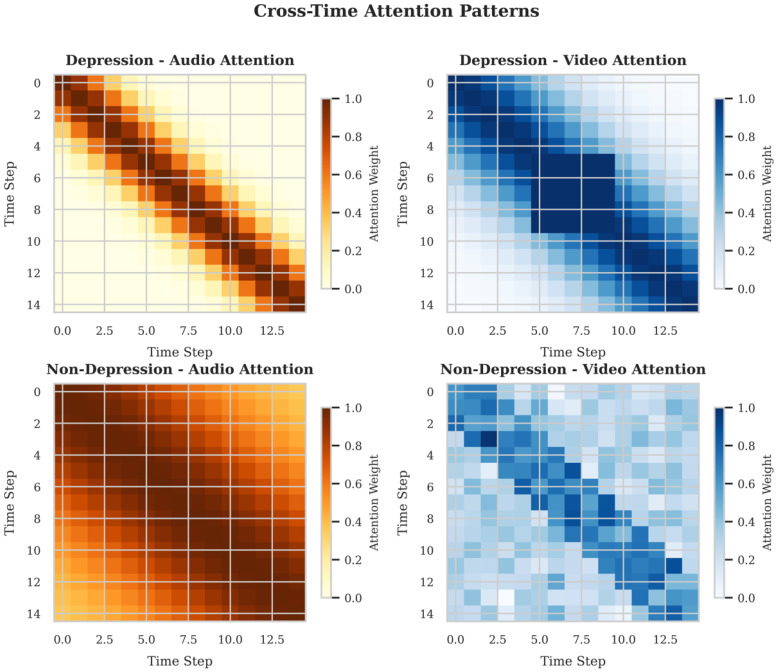
Cross-Time Attention Patterns for Depression and Non-Depression Detection in Audio and Video Modalities. The color bars indicate the attention weights ranging from 0.0 to 1.0. Each row in the attention maps is normalized such that the sum of the weights equals 1 (*N* denotes analyzed temporal segments).

Figure 9 illustrates the cross-time attention patterns of the model in the audio and video modalities for both depressed and non-depressed samples. In depression detection, the audio attention map exhibits a diagonal distribution, indicating that the model primarily focuses on the speech correlations between adjacent time steps, capturing short-term dependency features in the speech of depressed individuals. In contrast, the video attention for depressed individuals is concentrated in the middle time period (steps 5 to 10), suggesting that facial expressions or behaviors at specific moments are crucial for depression diagnosis.

For non-depressed individuals, the audio attention is more evenly distributed across time steps, reflecting the need for a comprehensive time-series analysis of speech features in healthy individuals. The video attention for non-depressed individuals, however, is more dispersed, with no clear key time points, indicating that their visual behavior features are more diverse. Through these differentiated attention patterns, the model effectively captures behavioral features of depression from the time-series data, providing an interpretable diagnostic basis for multimodal depression detection.

#### 5.4.3. Model Performance Under Different Noise Levels (RQ4)

**Figure 10:**
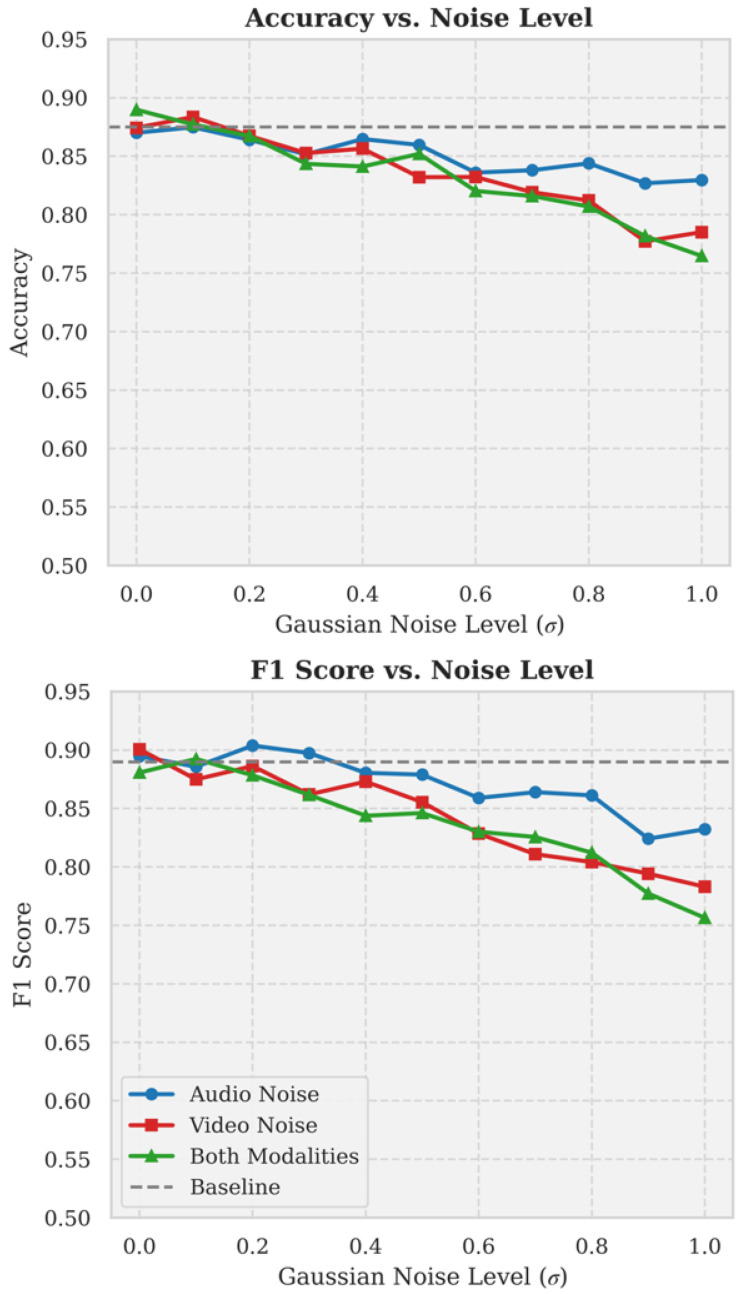
Performance Metrics of the Model under Varying Noise Levels(Error bars denote SD; *N* denotes test segments).

Figure 10 illustrates the model’s performance under varying levels of noise, specifically showing the changes in accuracy and F1 score when Gaussian noise is added to audio, video, or both modalities simultaneously. The upper plot demonstrates the change in accuracy with increasing noise levels: the blue line represents audio noise, where accuracy decreases gradually; the red line represents video noise, where accuracy drops more significantly; the green line indicates the combined effect of audio and video noise, where accuracy decreases most sharply. The lower plot depicts the trend in F1 score: the blue line shows that audio noise has a minimal impact on the F1 score; the red line indicates a larger impact from video noise; and the green line illustrates a substantial decrease in F1 score when both audio and video noise are present. At the highest noise level (σ=1.0), the F1 score drops to approximately 0.75.

The results suggest that the model is most robust to audio noise, followed by video noise, with the combined effect of both modalities having the most significant impact on model performance. At higher noise levels (σ>0.6), there is a notable decline in performance. Nonetheless, even at the highest noise level, the model’s accuracy and F1 score remain at relatively acceptable levels (>0.75), indicating a certain degree of robustness. Notably, when video noise has a larger impact, the model appears to rely more heavily on video features for decision-making. The performance decline under the combined effect of audio and video noise is not a simple additive effect, suggesting that the model can effectively compensate between modalities. While environmental noise does affect model performance, in practical applications—particularly when one modality is disrupted—the model can still maintain high performance, especially when the other modality provides complementary information.

#### 5.4.4. Sensitivity Analysis of Imbalanced Data Handling Strategies

**Figure 11:**
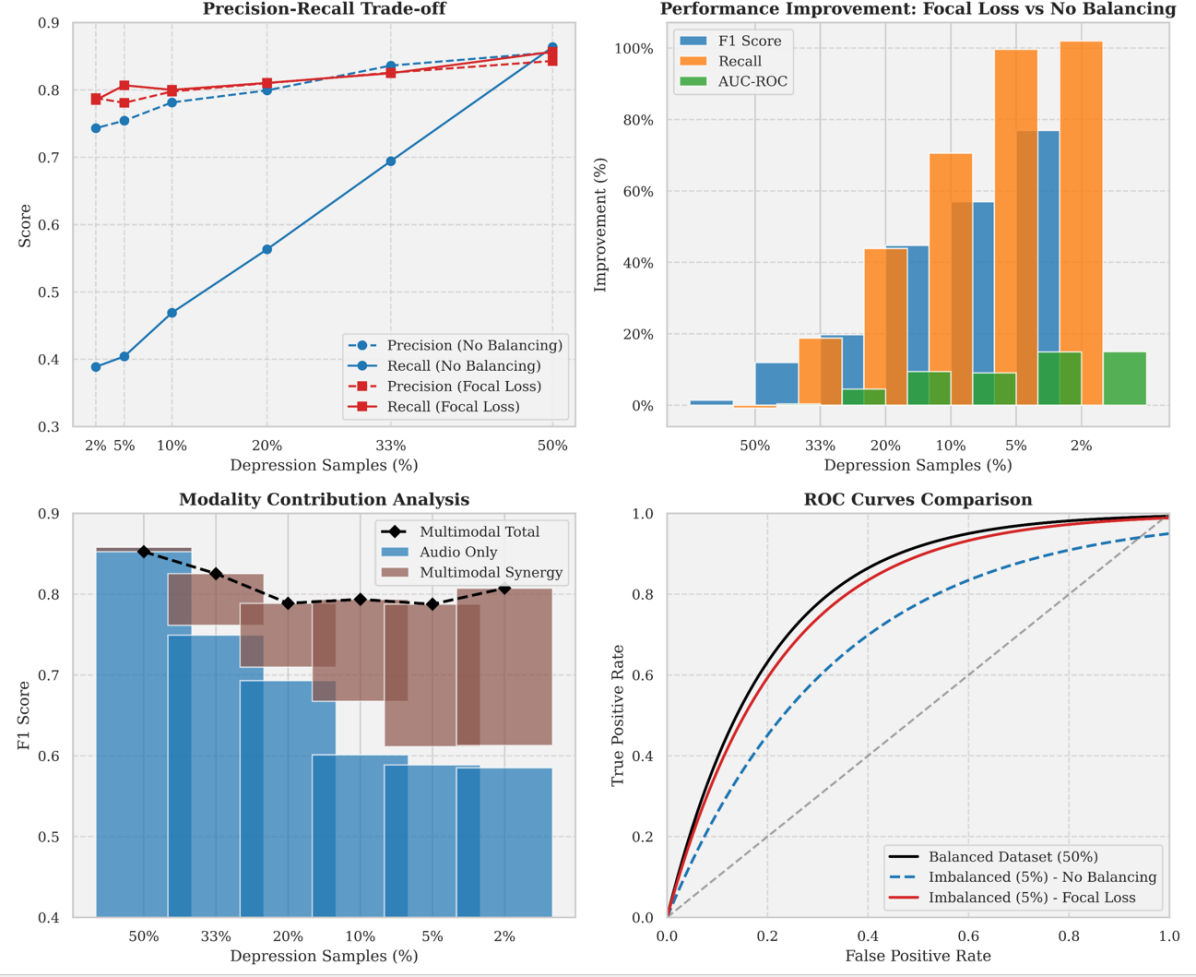
Performance Comparison under Different Balancing Strategies (N=8 representative cases).

Figure 11 presents a sensitivity analysis on an imbalanced dataset, comprising four subplots that analyze the model’s performance and the effects of different handling strategies in the context of data imbalance.

- Precision-Recall Tradeoff (Top Left): As the proportion of depressed samples decreases from 50% to 2%, without any balancing strategy (blue line), the recall rate declines significantly (from 0.85 to 0.4), indicating a reduced ability of the model to detect the minority class. In contrast, when using the Focal Loss strategy (red line), even under extreme imbalance conditions (2%), the recall rate remains high (approximately 0.8), with only a small change in precision and stable false positive rates.
- Performance Improvement Percentage (Top Right): Compared to the baseline with no balancing strategy, Focal Loss shows a more significant performance improvement as data imbalance intensifies. Under the extreme imbalance condition of 2% depressed samples, recall improves by over 100%, and the F1 score increases by approximately 70–80%, demonstrating the effectiveness of Focal Loss in highly imbalanced data.
- Modality Contribution Analysis (Bottom Left): As data imbalance increases, the contribution of different modalities to the model’s performance changes. The performance of the audio modality declines rapidly, while multimodal fusion effectively enhances model performance under high imbalance, maintaining a relatively stable F1 score.
- ROC Curve Comparison (Bottom Right): On the 50% balanced dataset (black line), the model performs the best. The Focal Loss strategy (red line) significantly outperforms the no-balancing strategy (blue dashed line) under the 5% imbalance condition, nearly recovering the performance of the balanced dataset.

The Focal Loss strategy effectively addresses the issue of data imbalance, while multimodal fusion further enhances the model’s robustness. This strategy is particularly important for clinical applications, as in real-world scenarios, depression samples are often the minority class, requiring specialized strategies to ensure detection performance.

#### 5.4.5. Robustness of Multimodal Models Under Temporal Disturbances

**Figure 12:**
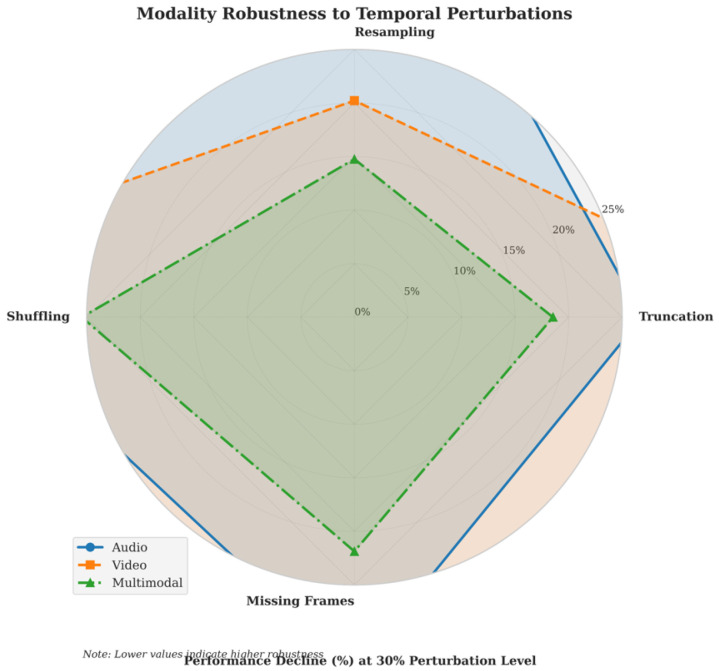
Performance Comparison under Different Temporal Disturbances(Error bars denote SD; *N* denotes experimental runs).

Figure 12 illustrates the robustness of the multimodal model under different types of temporal disturbances. The radar chart includes three modalities: audio (blue), video (orange), and multimodal (green), as well as four types of disturbances: truncation, resampling, shuffling, and missing values. The values represent the percentage of performance degradation, with lower values indicating stronger robustness against the disturbances.

The multimodal (green) model exhibits the best robustness, with the least performance degradation across all disturbance types. The audio modality (blue) is most sensitive to resampling, with a performance degradation of approximately 40%. The video modality (orange) performs poorly under sequence shuffling but is relatively robust to resampling. The impact of missing values on all modalities is minimal.

The area formed by the green multimodal curve is the smallest, indicating that multimodal fusion significantly enhances the model’s resistance to disturbances, with only a 10–15% decrease in performance. Audio is most sensitive to resampling, while video performs poorly under sequence shuffling. Resampling has the greatest impact on single modalities, while shuffling and truncation have a more moderate effect.

#### 5.4.6. Analysis of Modality Missing Experiment Results (RQ5)

**Figure 13:**
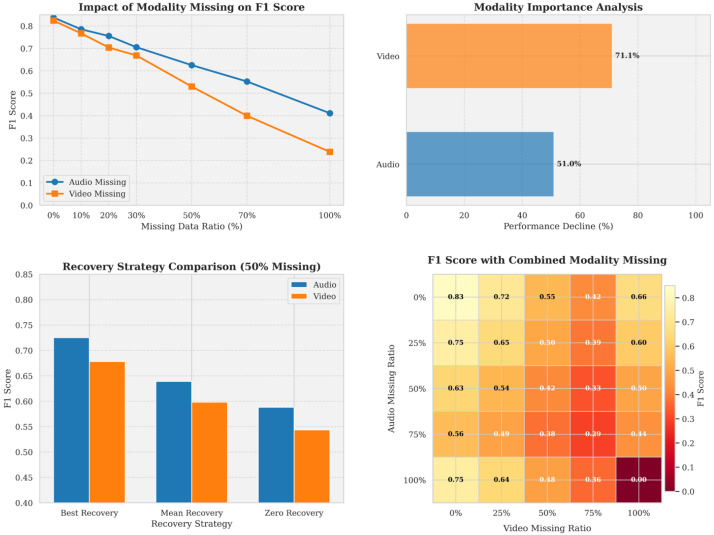
Effect of Modality Missing and Recovery Strategies(*N* denotes visualized samples).

Figure 13 presents the results of the modality missing experiment, consisting of four subplots. The upper-left plot shows the impact of different modality missing rates on the F1 score. The upper-right plot presents the modality importance analysis. The lower-left plot compares various recovery strategies (50% missing), and the lower-right plot displays a heatmap of F1 scores under joint modality missing conditions.

- The upper-left plot illustrates the effect of modality missing on the F1 score at various missing rates for audio and video modalities. It is evident that missing video modality (orange line) leads to a rapid decline in the F1 score, particularly when the missing rate exceeds 30%. In contrast, missing the audio modality (blue line) has a more moderate impact, suggesting that video features contribute more significantly to the model’s performance.
- The upper-right plot visually demonstrates the importance of the two modalities. The complete absence of the video modality results in a 71.1% performance degradation, while the absence of the audio modality only leads to a 51.0% drop in performance. This further confirms the higher importance of the video modality in depression detection.
- The lower-left plot compares three different data recovery strategies: optimal recovery, mean imputation, and zero imputation. Regardless of the modality, the optimal recovery strategy (e.g., using GANs or autoencoders) achieves the highest F1 score. Additionally, for all recovery strategies, the recovery performance of the audio modality outperforms that of the video modality, consistent with the greater complexity of video data.
- The lower-right plot presents a heatmap of the F1 scores for the audio and video modalities at various missing rates. The brighter the color, the higher the F1 score. It is observed that when no modality is missing, the F1 score is highest (0.83). When one modality is entirely missing (100%), the integrity of the remaining modality becomes crucial. When both modalities are severely missing (lower-right corner), the model’s performance significantly declines. The interactive pattern in the heatmap indicates that there is a synergistic effect between modalities, where the integrity of one modality can partially compensate for the loss of the other modality.

These plots illustrate the robustness of the multimodal depression detection model to missing data and emphasize the critical role of the video modality in depression detection. They also suggest that appropriate data recovery strategies can effectively mitigate the performance degradation caused by modality missing.

#### 5.4.7. Analysis of Computation Paths and Efficiency in DynMM Model

**Figure 14:**
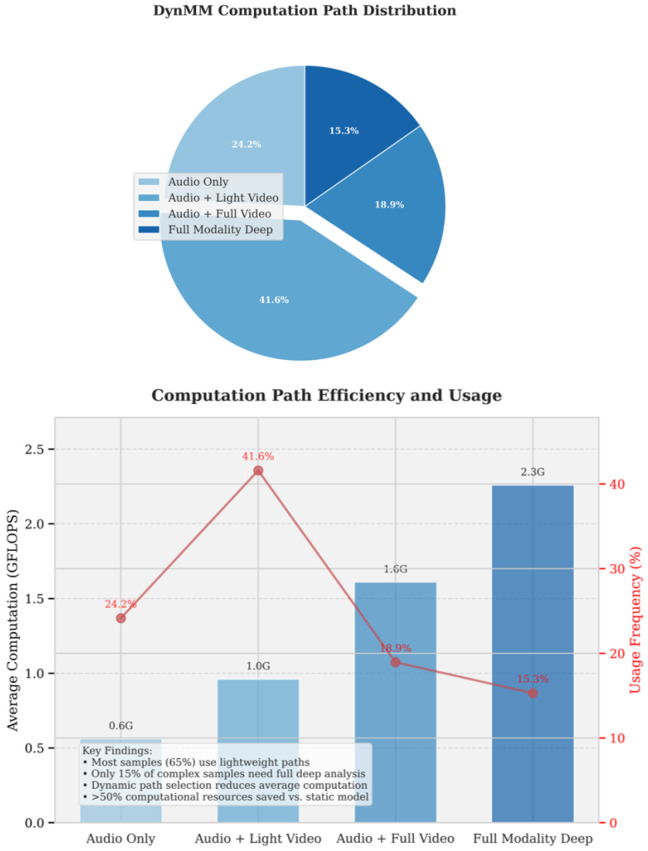
Computation Path Distribution and Efficiency in DynMM Model.

Figure 14 presents the distribution and efficiency of computational paths in the DynMM model, comprising two parts: the upper section shows the distribution of computational paths (pie chart), while the lower section illustrates the efficiency and usage frequency of these paths (bar chart and line graph).

The upper pie chart displays the usage proportions of the four computational paths. The audio + lightweight video path is the most frequently used, accounting for 41.6%. The audio-only path accounts for 24.2%, the audio + full video path for 18.9%, and the full-modal deep analysis path for only 15.3%. This indicates that most tasks opt for lighter computational paths.

The lower bar chart presents the average computational load (GFLOPS) for each path, ranked from lightest to heaviest: audio-only (0.6 G), audio + lightweight video (1.0 G), audio + full video (1.6 G), and full-modal deep analysis (2.3 G). The line graph shows the usage frequency of each path, which exhibits an inverse relationship with computational load: the audio + lightweight video path is the most frequently used (41.6%), followed by audio-only (24.2%), audio + full video (18.9%), and full-modal deep analysis (15.3%).

The annotation box in the lower-left corner summarizes the key findings: approximately 65% of the samples are processed using lightweight paths, while only 15% of the more complex samples require full-modal deep analysis. Dynamic path selection significantly reduces computational load, saving over 50% of computational resources compared to static models.

The DynMM model intelligently balances computational efficiency and model performance through dynamic path selection. Lightweight paths satisfy the majority of tasks, while heavier paths are reserved for a small subset of complex samples, making DynMM highly efficient during inference, particularly in resource-constrained application scenarios.

### 5.5. Error Analysis and Case Study

To further evaluate the robustness of DynMultiDep, we conducted a qualitative analysis of misclassified samples from the D-Vlog and LMVD datasets. Table 7 summarizes eight representative failure cases, categorized by their primary modality and underlying cause. The analysis reveals three main challenges: (i) **Visual Dynamics:** Rapid head movements (S2) and occlusions (S1, S8) disrupt the facial landmark tracking, leading to fragmented temporal features. (ii) **Acoustic Interference**: Non-depressive physiological factors, such as vocal hoarseness from a cold (S3) or excessive environmental noise (S5), can mislead the gating network. (iii) **Atypical Presentation**: Cases of “smiling depression” (S7) present a significant challenge, as their outward non-verbal cues contrast with their underlying mental state. These findings suggest that future iterations should incorporate more robust de-noising algorithms and multi-view visual encoders to better handle complex real-world scenarios.

## 6. Conclusions

The proposed DynMultiDep framework integrates a Mamba–Transformer expert model with the dynamic multimodal fusion strategy DynMM, enabling adaptive modeling of the multidimensional characteristics of depression. Experimental results indicate that, compared with unimodal approaches, DynMultiDep captures multimodal information in a more comprehensive manner. In contrast to static multimodal methods, DynMM improves computational efficiency and model generalization by dynamically selecting modalities and adjusting information fusion strategies. These findings are largely consistent with prior studies on multimodal emotion recognition and depression detection. Nevertheless, performance fluctuations are observed under conditions of modality absence or data redundancy, suggesting that further improvements in robustness are required when handling complex and non-ideal data scenarios.

The datasets used in this study were obtained through publicly available links, which introduces certain limitations. In particular, the model’s generalization ability may be constrained in scenarios or populations not represented in the available data. Moreover, cultural differences, demographic factors, and the limited set of modalities considered may influence the model’s effectiveness across diverse groups. Consequently, the results should be interpreted with caution when extending the approach to cross-cultural or cross-modal applications.

From an application perspective, DynMultiDep has the potential to be integrated into remote mental health monitoring systems, early screening tools, or edge-based platforms to support real-time depression detection. However, practical deployment must carefully consider ethical risks, and it should be clearly emphasized that the proposed framework is intended for screening purposes only and should not be regarded as a substitute for formal clinical diagnosis.

Future work will investigate data balancing strategies, the incorporation of additional modalities (such as textual and behavioral data), and computational optimization techniques, including model quantization and lightweight attention mechanisms, to further enhance performance and applicability. Overall, while DynMultiDep demonstrates promising potential for multimodal depression detection within the current experimental scope, claims regarding its real-world impact should remain cautious and proportional, grounded in empirical evidence rather than overstated generalizations.

## Figures and Tables

**Table 1:** Depression Detection Performance on the D-Vlog Dataset (N=961 subjects; Mean ± SD). The proposed method, DynMultiDep, is presented in the rightmost column. Bold values indicate the best performance across all comparative methods.

Modal Features	Metric	Transformer	LSTM	BiLSTM	GRU	TCN	ResNet	MMDNet	DynMultiDep
A+V	Accuracy	0.6564	0.5849	0.6447	0.5802	0.6226	0.5802	0.6604	**0.8000**
	Precision	0.6667	0.5956	0.6778	0.5802	0.6188	0.5810	0.6783	**0.7917**
	Recall	0.8687	0.8862	0.7534	1.0000	0.9106	0.9919	0.7886	0.8906
	F1 Score	0.7544	0.7124	0.7110	0.7343	0.7368	0.7327	0.7293	**0.8382**
A (Audio)	Accuracy	0.6038	0.5802	0.5802	0.5595	0.5802	0.5991	0.6415	**0.7692**
	Precision	0.6211	0.5802	0.5802	0.5776	0.5802	0.5990	0.6407	**0.7284**
	Recall	0.8130	1.0000	1.0000	0.9116	1.0000	0.9350	0.8699	0.9672
	F1 Score	0.7042	0.7343	0.7343	0.7071	0.7343	0.7302	0.7379	**0.8310**
V (Visual)	Accuracy	0.5613	0.5849	0.5755	0.5896	0.6375	0.6032	0.5802	**0.7745**
	Precision	0.5949	0.5936	0.5782	0.6023	0.6316	0.6035	0.5817	**0.7701**
	Recall	0.7642	0.9024	0.9919	0.8618	0.9796	0.9320	0.9837	0.9571
	F1 Score	0.6690	0.7161	0.7305	0.7090	0.7680	0.7326	0.7311	**0.8535**

**Table 2:** Depression Detection Performance on the LMVD Dataset (N=254 subjects; Mean ± SD). The proposed method, DynMultiDep, is presented in the rightmost column. Bold values indicate the best performance across all comparative methods.

Modal Features	Metric	Transformer	LSTM	BiLSTM	GRU	TCN	ResNet	MMDNet	DynMultiDep
A+V	Accuracy	0.6995	0.7213	0.6685	0.7104	0.6995	0.7219	0.7268	**0.7651**
	Precision	0.6667	0.7564	0.6581	0.7111	0.7045	0.6970	0.7356	**0.7586**
	Recall	0.7912	0.6484	0.7033	0.7033	0.6813	0.6765	0.7033	**0.8250**
	F1 Score	0.7236	0.6982	0.6783	0.7072	0.6927	0.6441	0.7191	**0.7904**
A (Audio)	Accuracy	0.5410	0.5574	0.5355	0.6099	0.5738	0.5191	0.5410	**0.6600**
	Precision	0.5229	0.5362	0.5190	0.5714	0.5455	0.5090	0.5238	**0.6790**
	Recall	0.8791	0.8132	0.9011	0.9014	0.8571	0.9341	0.8462	0.6875
	F1 Score	0.6557	0.6463	0.6586	0.6995	0.6667	0.6589	0.6471	0.6832
V (Visual)	Accuracy	0.6612	0.5660	0.6612	0.6721	0.6831	0.6721	0.7377	**0.7452**
	Precision	0.6355	0.5771	0.6381	0.6240	0.6602	0.6505	0.7263	**0.7416**
	Recall	0.7473	0.9431	0.7363	0.8571	0.7473	0.7363	0.7582	0.7952
	F1 Score	0.6869	0.7160	0.6837	0.7222	0.7010	0.6907	0.7419	**0.7674**

**Table 3:** Comparison with State-of-the-Art Methods on the D-vlog Dataset (N=961 subjects; Mean ± SD). The proposed DynMultiDep is compared against various baselines, with bold values indicating the superior performance of our method in each comparison group.

Methods	Accuracy	Precision	Recall	F1 Score	UA	WF1
MCRVT	63.1	72.51	67.74	70.03	62.53	54.1
DynMultiDep	**65.4**	**75.15**	**70.21**	**72.58**	**64.81**	**56.07**
Spike Memory Transformer	70.73	\	\	\	\	\
DynMultiDep	**71.83**	\	\	\	\	\
JAMFN	\	68.18	68.39	68.25	\	\
DynMultiDep	\	**69.18**	**69.24**	**69.38**	\	\
DepMamba	67.87	67.2	85.73	75.33	\	\
DynMultiDep	**80**	**79.17**	**89.06**	**83.82**	\	\

**Table 4:** Comparison with State-of-the-Art Methods on the LMVD Dataset (N=254 subjects; Mean ± SD). The best results for each metric are highlighted in bold, demonstrating the effectiveness of the proposed DynMultiDep.

Method	Accuracy	Precision	Recall	F1 Score
DepMamba	70.13	68.24	74.44	71.17
LMTformer	74.22	69.96	82.24	75.62
DynMultiDep	**76.51**	**75.86**	**82.50**	**79.04**

**Table 5:** The statistical significance analysis of our model compared to the other seven baseline models on the D-Vlog dataset (all baseline methods are marked with ***, indicating *p*-values less than 0.001). Mean Diff refers to the mean deviation (Mean Difference), indicating the average absolute deviation of values from the mean, and is a statistical measure.

Baseline	Mean Diff	Effect Size (Cohen’s d)	Adjusted *p*-Value	Significance
Transformer	0.08982	5.156104	$4.780833\times 10^{-13}$	***
LSTM	0.063626	3.293931	$1.280016\times 10^{-8}$	***
BiLSTM	0.110838	5.399268	$1.538949\times 10^{-13}$	***
GRU	0.072973	3.185507	$2.544152\times 10^{-8}$	***
TCN	0.088799	3.597811	$1.977933\times 10^{-9}$	***
ResNet	0.064569	3.183101	$2.583555\times 10^{-8}$	***
MMDNet	0.061750	4.024491	$1.655639\times 10^{-10}$	***

**Table 6:** The statistical significance analysis of our model compared to the other seven baseline models on the LMVD dataset (all baseline methods are marked with ***, indicating *p*-values less than 0.001. Mean Diff refers to the mean difference between our model and the baseline, representing the performance gain).

Baseline	Mean Diff	Effect Size (Cohen’s d)	Adjusted *p*-Value	Significance
Transformer	0.15182	1.542	<0.001	***
LSTM	0.252626	3.914	$1.119\times 10^{-23}$	***
BiLSTM	0.165838	1.725	$4.938\times 10^{-18}$	***
GRU	0.236973	3.382	$6.617\times 10^{-21}$	***
TCN	0.194799	2.246	$9.131\times 10^{-18}$	***
ResNet	0.234569	3.328	$3.225\times 10^{-22}$	***
MMDNet	0.164750	1.694	$2.414\times 10^{-21}$	***

**Table 7:** Summary of qualitative failure cases in DynMultiDep for depression detection. These samples represent typical scenarios where the gating network or feature extraction modules encountered challenges.

Case ID	Ground Truth	Prediction	Key Modality	Primary Reason for Misclassification
S1	Depressed	Healthy	Video	Severe facial occlusion (hands covering face)
S2	Depressed	Healthy	Video	Motion blur caused by rapid head rotation
S3	Healthy	Depressed	Audio	Vocal hoarseness due to seasonal cold/flu
S4	Depressed	Healthy	Audio	Extended silence segments exceeding 15 s
S5	Healthy	Depressed	Both	Significant environmental noise (street traffic)
S6	Depressed	Healthy	Video	Insufficient lighting masking facial micro-expressions
S7	Depressed	Healthy	Both	Atypical “smiling depression” with high masking
S8	Healthy	Depressed	Video	Heavy facial hair obstructing landmark detection

## Data Availability

The data presented in this study are openly available in GitHub at https://github.com/blzs-zhjk/DynMultiDep.
